# Versatility of Glutathione Transferase Proteins

**DOI:** 10.3390/biom13121749

**Published:** 2023-12-06

**Authors:** Bengt Mannervik

**Affiliations:** 1 Department of Biochemistry and Biophysics, Arrhenius Laboratories, Stockholm University, SE-10691 Stockholm, Sweden; bengt.mannervik@dbb.su.se; 2 Department of Chemistry, Scripps Research, La Jolla, CA 92037, USA

For more than 60 years, glutathione transferases (GSTs) have attracted attention, but the research field of the GSTome [1] has not yet matured. Originally discovered in the research on cellular protection against carcinogens and mutagens [2], GSTs were, by contrast, in cancer cells found to protect tumors against anti-cancer drugs. The biotransformation of other drugs was also established, in many cases following metabolic activation by cytochrome P450 enzymes. It was further concluded that a crucial GST function was the inactivation of genotoxic electrophiles such as unsaturated aldehydes formed in endogenous processes [3]. The engineering of GSTs for biotechnical applications followed suit [4]. Since then, new aspects have emerged every few years, and a full understanding of the scope of the GST proteins is not yet in sight.

The human genome encodes 17 GST proteins in seven classes, which are expressed as the “canonical” or cytosolic GST enzymes. In addition, homologous sequences forming chloride channels [5] or having other functions are known. Oakley has extended the gene family to 39 members [6] by mining databases for sequences and protein structures by use of artificial intelligence algorithms; in particular, AlphaFold [7] and AlphaFold-Multimer [8]. With the exception of prostaglandin E synthase 2 and the previously characterized GSTs, the new homologous proteins were not recognized as enzymes but were associated with other molecular activities. Oakley’s analysis also predicted that the dimeric canonical GST enzymes could form heterodimers, including subunits from different classes. However, the natural occurrence of inter-class hybrids is so far lacking experimental evidence, even though the existence of intra-class heterodimers is well-documented [9].

The review by Mazari et al. [10] addresses the role of GSTs related to diseases, with particular emphasis on cancer. In addition to protection against toxicants and a role in drug resistance [11], the interactions with other proteins and their functions in cellular signaling is indicated. The glutathionylation of SH groups in proteins is considered a posttranslational modification of biochemical significance. Townsend and Tew have forwarded GST P1-1 as a catalyst of this thiol–disulfide interchange. Recent findings reporting increased severity of SARS-CoV-2 (COVID-19) infections in GST-null individuals, lacking the *GSTM1* or *GSTT1* genes, are also reviewed.

A search for GST P1-1 inhibitors for possible use in overcoming anti-cancer drug resistance was published by Kupreienko et al. [12]. The crystal structure of *Mus musculus* GST P1-1 was solved, and a library of registered pesticides was screened for possible repurposing as inhibitors of the enzyme. The most potent compound found was the fungicide iprodione, which was observed intercalated between the active-site residues Phe8 and Tyr108 (with the authors’ numbering omitting the initiator Met1). Iprodione shows minimal toxicity to mammals and may therefore serve as a lead in the search for more potent inhibitors.

Russell and Richardson review the role of GSTs in nitric oxide (NO) metabolism [13]. The enzymes contribute to the release of the pharmacologically active NO from various drugs including the classical angina medicine nitroglycerin [14]. These drugs have applications as anti-cancer agents and as vasodilators for clinical use [15]. The GST proteins can also serve in the cellular storage of NO in the form of a stable dinitrosyl–iron complex with glutathione [16].

Scian et al. [17] propose that GST A4-4 plays a role as an enzyme regulating the cellular steady-state levels of lipid alkenals such as 4-hydroxynonenal. These products of lipid peroxidation have an important function as intracellular “second messengers” that contribute to homeostasis by managing the Nrf2-mediated phase-2 response involving the expression of anti-oxidant enzymes. High concentrations of the alkenals are cytotoxic and are counteracted by glutathione conjugation, followed by the exporting of the conjugate from the cell [18]. The conjugation reaction is reversible, and the backward direction safeguards an adequate fraction of unconjugated alkenal for regulatory functions. GST A4-4 efficiently catalyzes both forward and backward reactions and thereby makes sure that the alkenals are not completely eliminated by unidirectional detoxication.

Aldehydes and other low-molecular-mass molecules are among the odorous compounds that bind to GSTs and react with glutathione if they carry suitable functional groups. Schwartz et al. [19] are summarizing the evidence indicating that GSTs present in the olfactory tissues provide protection against toxic compounds. In addition, the enzymes appear to play a pivotal role in chemoperception by rapidly degrading bound odorants, thereby terminating the signal and preparing the receptors for new inputs [20]. GSTs occur abundantly in mammals as well as in insects, and in the various species investigated, the enzymes appear to play similar roles in their olfactory systems. In mammals, alpha-, mu-, and pi-class GSTs are implicated, whereas in insects, delta- and epsilon-class members are engaged. Notably, the former mammalian GST classes do not occur in insects [21], and the latter insect GST classes are absent in mammals. Thus, the development of GST functions in chemosensory perception seems to be an example of convergent evolution, which is enabled by the versatility of the GST protein fold.

A novel class of canonical GSTs was discovered in cyanobacteria by Wiktelius and Stenberg and was named chi [22]. The photosynthesis carried out by cyanobacteria caused the Earth’s atmosphere to change from an anaerobic reducing state to an oxygen-containing environment >2 billion years ago. The novel oxidizing conditions necessitated the emergence of protective agents such as glutathione, and it is a reasonable assumption that the evolution of GST was first established in cyanobacteria [22]. Mocchetti et al. [23] have solved the crystal structure of the chi-class GST from the cyanobacterium *Synechocystis*, and furthermore, they have compared it with primary structures from other species. The chi-class proteins featured a well-conserved Ser-Arg-Ala-Ser amino acid motif in the active site of the N-terminal region, but a Ser residue appeared to be non-essential for catalytic activity, as indicated by mutagenesis of the *Synechocystis* GST. By contrast, Ser in a similar position of GSTs from other classes plays a role in stabilizing the thiolate form of glutathione in the catalyzed reaction. In the chi-class, the Ser sidechain interacts instead with the protein backbone. Overall, the *Synechocystis* structure was similar to the known canonical GSTs of different classes, although the length of its α4 and α5 helices in the core of the protein was diminished as a consequence of a shorter primary structure. A phylogenetic analysis identified several hundred homologs to the *Synechocystis* GST, of which 147 featured the characteristic Ser-Arg-Ala-Ser sequence.

A physiological role of GST A3-3 in steroid hormone biosynthesis in humans and a few other mammals has been identified, which involves the double-bond isomerization of 5-androsten-3,17-dione and 5-pregnen-3,20-dione, precursors of testosterone, estradiol, and progesterone [24]. Although man and horse feature the enzyme GST A3-3 with prominent ketosteroid isomerase activity, cow, rat, and mouse do not have a GST with a corresponding high isomerase activity. Hubert et al. have cloned mammalian GSTs homologous to GST A3-3 from a broader phylogenetic range [25] in order to further explore the occurrence of the high-steroid isomerase activity in the GSTome.

In insects, a high-ketosteroid isomerase activity contributed by an epsilon-class GST was furthermore discovered in the malaria-transmitting mosquito *Anopheles gambiae* [26]. The catalytic efficiency (k_cat_/K_m_ = 6.9 × 10^6^ M^−1^s^−1^) of the mosquito GSTE8 matches that of the most active mammalian GST A3-3. A corresponding GSTE14 in *Drosophila melanogaster* had previously been identified as one of several enzymes essential to the biosynthesis of the insect steroid hormone ecdysone [27,28]. The fruit fly mutant deficient in the enzyme was named *noppera-bo* and the GST was consequently called Noppera-bo (or Nobo). The mutant insect is not viable, since ecdysone is required for metamorphosis, including hatching, molting, pupation, and eclosion. The cognate substrate of GSTE8/Nobo is unknown, but the prominent activity discovered with 5-androsten-3,17-dione, a steroid not found in insects, suggests that a similar ketosteroid related to ecdysone may be the natural counterpart.

Nobo has been identified in the orders *Diptera* and *Lepidoptera* but is absent in other insects. Thus, the enzyme has been recognized as an ideal target in combatting insects carrying infectious diseases. Selective inhibitors of Nobo would serve as insect growth regulators that would not jeopardize bees and other insects of agricultural importance, which do not express Nobo. Ebihara and Niwa [29] have directed particular attention to inhibitors of Nobo from the yellow-fever vector *Aedes aegypti* and identified flavonoids with high potency. The strongest inhibitor (IC_50_ = 0.287 µM) was desmethylglycitein, and the insecticidal activity was verified experimentally with larvae from *A. aegypti* [30].

The Special Issue on the “Versatility of Glutathione Transferase Proteins” exemplifies various aspects of GST research ranging from human genes and disease to metabolism and insects. The cyanobacterial GSTs point to a vast field of unchartered enzymology in the realm of prokaryotes. Not represented here is the research on plant GSTs and membrane-associated GSTs.
